# When Exercising for Metabolic Health, the Work is Never Done, But HIIT Will Save You Time

**DOI:** 10.1210/clinem/dgaa451

**Published:** 2020-07-17

**Authors:** Joseph W Beals, Brandon D Kayser

**Affiliations:** Center for Human Nutrition, Washington University School of Medicine, St Louis, Missouri

Obesity is often associated with insulin-resistant glucose metabolism, and exercise training is a powerful tool for improving glucometabolic control. In fact, regular exercise is often regarded as a front-line treatment for improving insulin sensitivity in those with impaired glycemic control (1). The use of exercise as a therapeutic intervention is based on our well-established understanding of the effects of moderate-intensity continuous training (MICT). However, the need to improve population-wide exercise participation—and potentially the therapeutic benefit of exercise—has sparked greater research interest into the physiological effects of high-intensity interval training (HIIT), which aims to provide the same, or greater, benefits of MICT in less time by exercising with a much higher intensity over shorter exercise sessions. Indeed, the relative effectiveness of HIIT compared with MICT is starting to take shape.

The effect of HIIT versus MICT on traditional training adaptions has been studied in several populations with varying degrees of metabolic dysregulation. In populations with insulin resistance, for example, those with prediabetes or type 2 diabetes, it appears that HIIT may elicit a larger increase in aerobic fitness, measured by peak oxygen consumption during maximal exercise, compared with MICT (2). On the other hand, training-induced improvements in biomarkers of metabolic health, such as hemoglobin A_1c_ and low-density lipoprotein cholesterol (2), and oral glucose tolerance (3) have been shown to be largely independent of exercise intensity in adults with prediabetes. Previous studies comparing the effect of HIIT or MICT on glucose metabolism, however, have relied on indices or indirect measures of insulin sensitivity.

In a recent issue of this journal, Ryan et al (4) performed a comprehensive evaluation of multiorgan insulin sensitivity and related molecular pathways in adults with obesity who completed 12 weeks of HIIT or MICT, performed using either cycling, treadmill, elliptical, or rowing exercise modalities. The authors used the gold standard method for determining insulin sensitivity: a hyperinsulinemic-euglycemic clamp procedure combined with stable isotopically labeled metabolic tracers. Importantly, insulin sensitivity was determined before and then 1 and 4 days after the final exercise session, which allowed them to distinguish between the response to the last exercise session and the longer-term adaptions to exercise training. This study found that whole-body insulin sensitivity was improved similarly by both HIIT and MICT 1 day after exercise cessation. Despite this improvement the following day, whole-body insulin sensitivity in both groups had returned to pretraining values at 4 days after the final training session. Several key molecular aspects of skeletal muscle metabolism were also characterized, including content of glycogen and mitochondrial proteins, as well as lipidomics and the regulation of lipolysis, though all of these measurements changed similarly between MICT and HIIT groups and ultimately appeared unrelated to the changes in insulin sensitivity induced by exercise training.

A notable finding in this study is that several well-known skeletal muscle adaptions to exercise training (eg, increased mitochondrial proteins and lipid droplet enzymes) remained elevated 4 days after training in both training groups despite the reversal of the improvement in insulin sensitivity, which again, challenges the role they may have in mediating the insulin-sensitizing effects of exercise. Despite the similarities between HIIT and MICT seen in this study, it is prudent to consider that much of our current understanding of the health benefits of exercise is based on continuous moderate-intensity exercise. As such, longer-term studies focused on insulin sensitivity as well as other health benefits known to be derived from moderate-intensity exercise will be needed before HIIT can be deemed completely interchangeable with conventional exercise programs (5).

The findings of Ryan et al (4) also have practical implications: both HIIT and MICT lead to similar improvements in insulin sensitivity, and participants were allowed to choose their exercise modality, as would be the case in a real-world setting, which suggests that metabolic improvements can be obtained with a variety of cardiovascular training equipment. Using HIIT to reduce time commitment and allowing a variety of training modalities to choose from may be valuable tools for anyone struggling to balance schedules and priorities or who may otherwise want to add variety to an existing exercise plan. Finally, another important finding from this report is that the insulin-sensitizing effect of exercise, regardless of intensity, may be relatively short-lived. These findings recapitulate the notion that many of the cardiometabolic benefits of exercise are conferred in response to the most recent bout of exercise (6), which emphasizes the importance of continued participation in any exercise program. This is also supported by a recent meta-analysis conclusion that a variety of different training paradigms are capable of eliciting a metabolic benefit, provided that the exercise is conducted on a regular basis (7). Altogether, studies such as this are important to our understanding of the health benefits of HIIT and other less conventional exercise programs, which may lead to a greater incorporation of exercise into the daily routines of those who will benefit most, irrespective of its form.

## Data Availability

Data sharing is not applicable to this article as no datasets were generated or analyzed during the current study.

## Abbreviations

HIIT: high-intensity interval training
MICT: moderate-intensity continuous training

## Additional Information

***Disclosure Summary*:** The authors have nothing to disclose.
